# Notes and News

**Published:** 1892-06-25

**Authors:** 


### Notes and News.

Collected and Communicated.

This week the Prince of Wales contributed some roses from Sandringham for the Lady Mayoress's stall at the Rose Show held at the Mansion House on Friday, 24th inst., in aid of the Royal Hospital for Children and Women, in the Waterloo Road. The Princess Christian opened the fête, and was accompanied by her daughter, the Princess Victoria of Schleswig-Holstein.

We are glad to see that the Treasurer of the Chelsea Hospital for Women has adopted that form of appeal, namely, the letter-card, which we commended in a previous issue. We feel sure that the use of it by institutions will prove welcome to the recipient, and be a means of saving the hospital much expense and trouble. In the case of the Chelsea Hospital, Mr. Wright has made the best use of the space available to put forth the claims and advantages of his institution in a telling and concise manner, and has added a short personal appeal also.

The Ladywell Sanatorium at Salford, or hospital for infectious diseases, was opened on the 15th inst., at a cost of £65,000. It affords accommodation for 184 patients. Isolated blocks are provided for the purpose of doubtful cases; for the treatment of patients when only one or two are suffering from the same disease; and for the reception of delirious or otherwise undesirable inmates of the general wards. There are also private wards for paying patients. Eight wards accommodate three, and eight two patients in each. A nurses' duty room is connected with every two wards. The hospital has the advantage of a verandah running along the west side, appertaining to each floor. A special feature in the building is the height of the side windows of each ward, which extend from the boarding to the ceiling, without a break. Corners have been avoided in the construction, and a most complete system of warming and ventilation adopted. Space forbids us dwelling at greater length here on the further details of this excellent institution, which has just entered on a career of undoubted usefulness.

The Children's Hospital, which was opened at Cold Ash on the 8th inst., is a two-storey building, connected with the Children's Home by a long corridor, and capable of further extension. The ground floor ward has glass doors opening on the garden, that the cots may be removed thence when desirable. An isolation ward is so placed as to admit of complete separation from the rest of the building. There is admirable provision everywhere for lighting and ventilation. Two very cheerful rooms have been provided for nurses. Mr. James H. Money was entrusted with the preparation of the plans.

Amongst the many interesting entertainments to be held next week in aid of various London charities we especially note a concert, on June 27th, under the patronage of the Queen, in aid of the National Society for the Prevention of Cruelty to Children, at St. James's Hall. On the 28th Paddington Green Children's Hospital is to benefit by a matinée to be given at the Strand Theatre, when a new play, "Lady Brown's Diary," is to be produced. At the Grosvenor Club, on the same evening, a performance of Glück's "Orféo" is to take place, under the patronage of the Duke of Connaught. This performance will be repeated on the 30th inst.

The scheme pursued by the management of the Mothers' Seaside Convalescent Home at Herne Bay, of which the Bishop of London is President, is an excellent one. The institution receives women and girls who are convalescent, and need rest, change, and liberal diet, mothers being permitted to bring their infants with them. The nursery is quite away from the rest of the building, so that no annoyance is incurred. The Home has not been opened many months, but over 150 visitors have already been received. The charge is only 10s. 6d. per week for adults, and less for children. A "Provident Section" provides that "Any person whose income is £2 2s. or less per week may, by payments of not less than 1s. at convenient intervals, subscribe for the benefit of the family."

On Saturday last a charming concert was given at Grosvenor House by permission of the Duke of Westminster, the proceeds of which were to further the useful work of the Popular Musical Union. The choir and orchestra of the Union, numbering 120 performers, under Mr. Thomas, rendered the chorus of the several selections in excellent style, and Mr. Ben Davies, Madame Belle Cole, Miss Helen Trust, and others gave the solos, while Mr. Ganz conducted. Mrs. Ernest Hart, the Hon. Secretary, presented the ninth annual report, and in a short speech put forth clearly the work and success that the Union has achieved. We could only feel sympathy and admiration for the steady progress of a scheme which, starting amidst sneers, and cynically dubbed "Utopian," has ended in providing the best music to highly appreciative audiences in East and South London. If space permitted we should like to trace the movement from a single ballad introduced among ordinary vulgar music-hall ditties, to the present day, when an East-end audience may be found carefully following "Judas Maccabæus," score in hand. Nobody in their senses doubts the refining influence of real music, and we recommend doubters to go and see an East-end audience and then judge. Meanwhile, will our readers help on the choral and orchestral classes which the Union holds and send some subscription to Mr. Lionel R. Foot, 33, Brewer Street, Regent Street.

